# Evaluation of the clinical value of bone metabolic parameters for the screening of osseous metastases compared to bone scintigraphy

**DOI:** 10.1186/1471-2385-4-3

**Published:** 2004-12-04

**Authors:** Johann Schoenberger, Silke Rozeboom, Eva Wirthgen-Beyer, Christoph Eilles

**Affiliations:** 1 Department of Nuclear Medicine, University of Regensburg, Germany

## Abstract

**Background:**

Bone metastases are common in many types of cancer. As screening methods different imaging modalities are available. A new approach for the screening of osseous metastases represents the measurement of bone metabolic markers. Therefore aim of this study was to evaluate the usefulness of the determination of bone metabolic markers aminoterminal propeptide of type I procollagen (PINP, osteoblastic activity) and the carboxyterminal pyridinoline cross-linked telopeptide of type I collagen (ICTP, osteoclastic activity) for the detection of bone metastases associated with other malignancies.

**Methods:**

88 patients aged 21 – 82 years with malignant tumors were prospectively studied. The serum concentrations of PINP and ICTP were measured and compared to the results of bone scintigraphy, radiological bone series, CT, MRI and clinical follow-up.

**Results:**

Osseous metastases were found in 21 patients. 19 of them were correctly identified by bone scintigraphy (sensitivity: 90%). For bone metabolic markers results were as follows: ICTP sensitivity: 71%, specificity: 42%; PINP sensitivity: 24%, specificity: 96%.

**Conclusions:**

As markers of bone metabolism PINP and ICTP showed low sensitivity and/or specificity for the detection of osseous metastases. The presented markers did not seem to be sufficient enough to identify patients with bone metastases or to replace established screening methods.

## Background

Bone metastases are common in advanced cancers of the lung, breast, kidney, prostate and others. In autopsy studies the prevalence ranges from 47–85% [1]. In patients with osseous metastases early detection is needed, since without effective treatment these bone metastases can cause severe complications leading to considerable morbidity and reduced quality of life.

The screening for bone metastases is usually based on bone scintigraphy, confirmed by supplemental radiographic bone surveys, computer tomography, or magnetic resonance imaging. Bone scans are simple to perform and examine the whole skeleton, but although the sensitivity of this method is high its specificity is poor. For this reason, in many cases a positive scan requires confirmation by other imaging modalities, which leads to higher costs and time-consuming investigations. Therefore, an alternative cost-effective screening method with a similar sensitivity and a higher specificity would be very welcome.

For the formation of osseous metastases the extracellular matrix consisting of collagens combined with noncollagenous glycoproteins and proteoglycans, including the basement membrane and the interstitial stroma play an important role. Normally, the extracellular matrix serves as a barrier for the attachment and invasion of malignant cells, but the proteolytic activity of tumor cells leads to the destruction of its collagenic components, thus facilitating the local invasion of malignant cells and finally the development of bone metastases [2]. The major collagen in bone is type I collagen, which is synthesized by osteoblasts and accounts for about 90% of the organic matrix [3].

Recently, bone metabolic markers have been reported to be useful in diagnosing bone metastases [4,5]. Newly developed methods are able to quantitatively determine concentrations of collagen metabolites. The synthesis of type I collagen can be analyzed by measuring the serum concentration of the aminoterminal propeptide of type I procollagen (PINP) using a specific radioimmunoassay. In addition, bone resorption can also be analyzed by a radioimmunoassay, which measures the serum concentration of the carboxyterminal pyridinoline cross-linked telopeptide of type I collagen (ICTP).

Both parameters have been identified as potential candidates for the early detection of bone metastases. Therefore, the purpose of this study was to evaluate the usefulness of PINP and ICTP in patients with newly diagnosed cancer for the screening of osseous metastases compared to current standard protocols.

## Methods

### Patients

For this study 88 consecutive patients (35 female; 53 male) with malignant tumors (20 lung cancer, 20 breast cancer, 19 head/neck cancer, 5 prostate cancer, 4 thyroid cancer, 3 sarcoma, 2 esophagus cancer, 2 pancreatic cancer, 2 urothel cancer, 1 gastric cancer, 2 plasmocytoma, 2 histiocytosis X, 1 melanoma, 1 rectal cancer, 1 hypernephroma, 2 carcinoma of unknown primary, 1 breast cancer and hypernephroma) between the ages of 21 and 82 were included. None of these patients were under a tumor specific therapy or presented primary bone disease such as osteoporosis or Paget's disease, which could interfere with the results of this study. All patients gave written consent to participate in this prospective study, which was approved by the local ethics committee.

### Marker assays

Blood samples for measuring PINP and ICTP were collected on the same day as bone scintigraphy. Due to higher PINP values at night, all samples were taken early in the morning and stored at -20°C until assayed. Apparent hemolytic serum was excluded.

Serum concentrations of ICTP and PINP were measured by using commercially available RIA kits (both: Orion Diagnostica, Espoo, Finland). According to the kit description, the normal range of ICTP was 1.6–5.3 μg/l for females and 1.4–5.2 μg/l for males. For PINP the normal range was 19–102 μg/l for females and 21–78 μg/l for males.

### Bone scanning

Two double-head gamma cameras (ECAM duet and Bodyscan; Siemens, Erlangen, Germany) and a triple-head gamma camera (Multispect III, Siemens, Erlangen, Germany) were used for planar and tomographic (SPECT) bone scans, respectively. Low-energy, high-resolution collimators (1024 × 256 matrix) were used and data acquisition was started 2–4 hrs after intravenous injection of 550–700MBq Tc^99m^-DPD (3,3-Diphosphono-1,2-propandicarbonacid). SPECT acquisitions were performed from suspected regions (128 × 128 matrix; 64 steps; 150000–200000 counts/step; Butterworth filter; cut-off level 0.4). The total acquisition time ranged from 100 to 130 min for planar and SPECT scans. The bone-scanning procedure was performed in accordance with procedural guidelines published by the Society of Nuclear Medicine [6].

### Interpretation of bone scintigraphy

Two experienced nuclear medicine physicians interpreted planar bone and SPECT images. Initially, neither of the readers knew of the findings of the other or the results of other imaging modalities. Increased tracer-uptake located at joints or on the edge of vertebral bodies adjacent to disk spaces was interpreted as arthritis or osteophytes, respectively. Lesions were classified as fractures when they showed a typical linear and curved pattern e.g. adjacent lesions in the ribs. Multiple lesions of varying size, shape and intensity, elongated rib lesions or photopaenic areas (cold spots) were classified as osseous metastases or suspicious lesions, where further analysis or imaging methods were necessary. In general, interpretation was performed following the criteria described by Krasnow et al. [7]. Finally, any discrepant interpretations between the two readers were resolved by consensus. Patients were classified as having osseous metastases when other imaging modalities (radiographic, MRI, CT) or histological findings confirmed the diagnosis. Patients were classified as not having bone metastatic disease when no imaging technique or histological finding indicated osseous involvement. To reduce the possibility that bone metastases were not yet visible by the cited imaging methods, a clinical follow-up period of between 9–14 months was used as the gold standard in all patients. Follow-up consisted of a clinical examination, control of tumor markers, imaging techniques (e.g. computed tomography, MRI or plain radiographic), etc. Overall, follow-up was done in compliance with the guidelines for tumor patients published by the cancer society.

### Data analysis

Sensitivity and specificity were calculated. Values are expressed as mean ± standard deviation. Statistical analysis was performed using SPSS-Software version 10.0 (SPSS, Inc.). The Mann-Whitney U test was applied to compare concentrations of PINP and ICTP, respectively. A value of p < 0.05 was considered to be statistically significant. Furthermore, we performed a receiver operating characteristic (ROC) analysis to assess the impact of bone metabolic markers in detecting bone metastases.

## Results

Osseous metastases were found in 21 patients with the following tumors: 8 patients with breast cancer, 3 patients with head/neck cancer and local osseous tumor invasion, 3 prostatic cancer, 2 plasmocytoma, 1 each lung cancer, histiocytosis X, thyroid cancer and sarcoma and 1 patient had breast cancer and hypernephroma.

### Bone scintigraphy

Using planar and tomographic bone scintigraphy, 19 patients were correctly diagnosed as patients with osseous metastases by both investigators independently and confirmed by other imaging modalities or follow-up (sensitivity: 90%). Two patients were false negative, 1 patient with histiocytosis X (diffuse infiltration of the spine and pelvis, identified by computed tomography) and 1 had breast cancer and hypernephroma (multiple osteolytic lesions with diameters up to 1.5 cm in the pelvic bone, which was correctly diagnosed by computed tomography). In 43 patients there was no evidence for osseous metastases by bone scintigraphy or other imaging modalities. In 24 patients, changes in bone scintigraphy were described not typical for osseous metastases, but additional imaging methods were recommended for verification. Most of these lesions were located in the ribs (singular focus) and the spine, typically for fractures, traumatic injuries or osteoporotic changes (compression fracture). Additional verification was recommended especially in those patients where no history of traumatic injuries, degenerative processes or osteoporosis was known. Verification was done in most cases by plain radiography, computed tomography or within the staging by FDG-PET.

### ICTP

**Figure 1:**
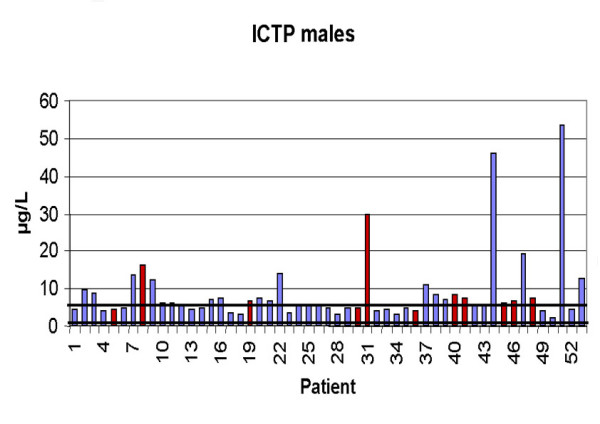
Values of ICTP in males (Reference interval: 1.4–5.2 μg/). Red columns indicate patients with osseous metastases.

**Figure 2:**
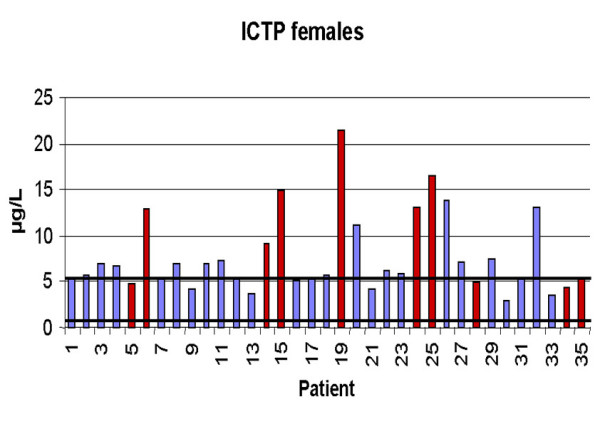
Values of ICTP in females (Reference interval: 1.6–5.3 μg/). Red columns indicate patients with osseous metastases.

Serum ICTP was elevated above the upper reference limit (>5.2 μg/ in males and >5.3 μg/l in females) in 56 patients (31 male, 25 female). In females the mean value was 7.64 ± 4.25 μg/l. In the group with metastases (n = 10) the mean value was 10.71 ± 5.90 μg/l compared to 6.41 ± 2.66 μg/l in the other group (n = 25) without metastases (p = 0.11). In males the mean value was 8.74 ± 9.49. The patients with metastases (n = 11) showed a value of 9.23 ± 7.62 compared to 8.61 ± 9.99 in patients (n = 42) without bone metastases (p = 0.24). Figures 1 and 2 present the values of all patients, separated by male and female, showing the different range of normal values. In females sensitivity was 70% and specificity 32% and in males sensitivity was 73% and specificity 48%. For both groups combined sensitivity was 71% and specificity 42%.

### PINP

**Figure 3:**
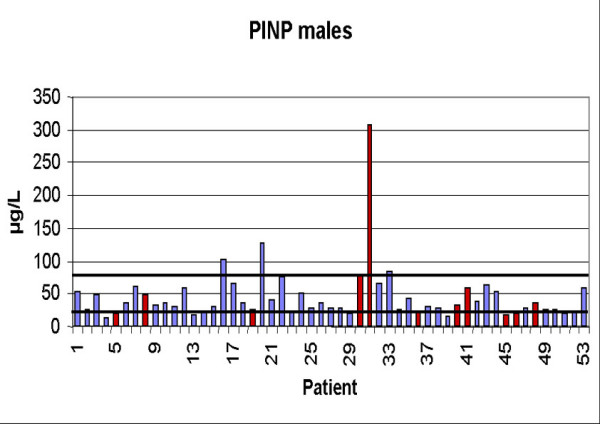
Values of PINP in males (Reference interval: 21–78 μg/). Red columns indicate patients with osseous metastases.

**Figure 4:**
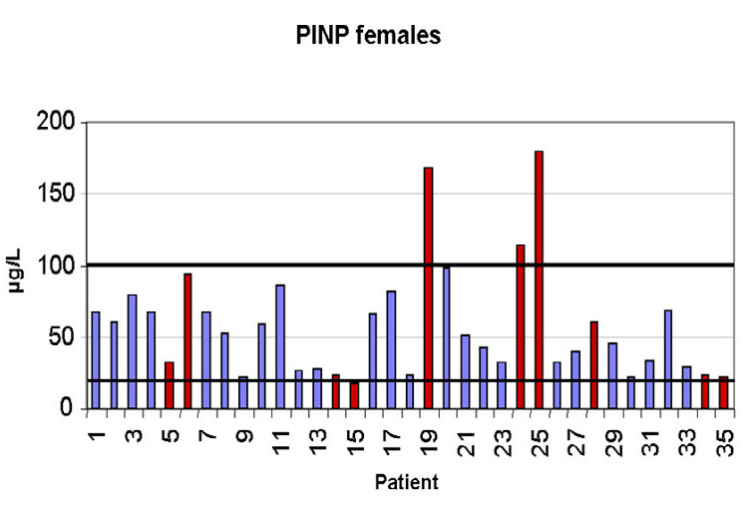
Values of ICTP in females (Reference interval: 19–102 μg/). Red columns indicate patients with osseous metastases.

Serum PINP was elevated above the upper reference limit (>78 μg/ in males and >102 μg/l in females) in 8 patients (5 male, 3 female). In females the mean value was 57.42 ± 38.50 μg/l. In the group of patients with metastases (n = 10) the mean value was 73.29 ± 62.38 μg/l compared to 51.07 ± 22.23 μg/l in the group (n = 25) without metastases (p = 0.95). In males the mean value was 45.59 ± 43.20. The patients with metastases showed a value of 60.70 ± 83.86 compared to 41.63 ± 23.99 in patients without bone metastases (p = 0.70). Figures 3 and 4 show the values of all patients. Sensitivity in females was 30 %, specificity 100 %, in males sensitivity was 18 %, specificity 93 %. Combined sensitivity was 24 % and specificity 96 %. As examples, figures 6 and 7 show patients with non-small-cell-lung cancer as the primary tumor.

### ROC-analysis

Neither parameter achieved statistical significance by ROC analysis. According to the ROC analysis, the optimal cut-off level of ICTP that maximizes sensitivity and specificity was 7.5 μg/l in females (60% sensitivity and 88% specificity) and 5.9 μg/l in males (73% sensitivity and 57% specificity), for PINP 85.5 μg/l in females (40% sensitivity and 96% specificity) and 25.8 μg/l in males (46% sensitivity and 74% specificity).

**Figure 5:**
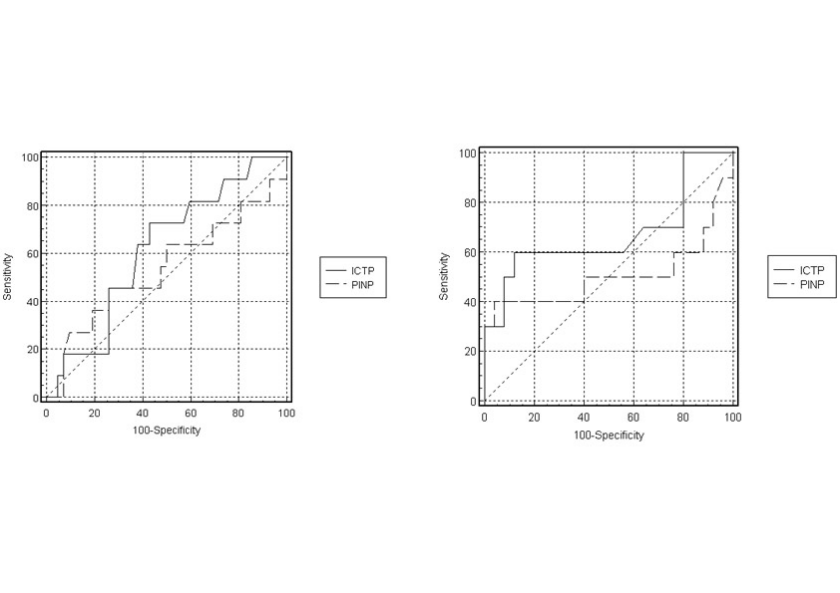
left side ROC curves for ICTP and PINP in males Marker AUC ± SE (95%CI) ICTP 0.62 ± 0.1 (0.47–0.75) PINP 0.54 ± 0.1 (0.40–0.68) AUC: area under curve; SE: standard error; 95%CI: 95% confidence interval  right side ROC curves for ICTP and PINP in females Marker AUC ± SE (95%CI) ICTP 0.67 ± 0.1 (0.49–0.82) PINP 0.51 ± 0.1 (0.33–0.68) AUC:area under curve; SE: standard error; 95%CI: 95% confidence interval

Figure 5 shows ROC curves and summarizes the results.

The types of tumors in our study were very heterogeneous, which reflects the normal day-to-day situation seen in a department of nuclear medicine. To obtain additional information concerning special tumor types, we performed a separate examination of two different tumor types with a higher number of patients: breast cancer and head/neck cancer.

### Breast cancer

The group with breast cancer consisted of 20 patients. Subsequently, bone metastases could be verified in eight of these patients. Six cases could be identified clearly by bone scan, in one patient additional plain radiographic analysis was recommended to confirm the diagnosis and one patient was false positive (focal area of increased tracer uptake in the shaft of the femur, identified as local necrosis of the bone by plain radiography and confirmed by follow-up). For bone markers the following results were observed:

ICTP: sensitivity: 63%, specificity: 25%. PINP: sensitivity: 25%, specificity: 100%. Figure 8 shows an example of a patient with osseous metastatic disease and normal values for ICTP and PINP.

### Head and neck cancer

19 patients with head/neck cancer were examined (4 female, 15 male). In these patients determination of whether local osseous structures are involved is essential for the preoperative planning of further treatment. In three patients osseous structures were affected by the tumor and all of these were correctly diagnosed by bone scintigraphy. ICTP and PINP were right positive in two separate patients. Sensitivity for PINP and ICTP was 33%, specificity for ICTP was 56% and for PINP 94%.

## Discussion

Metastatic bone disease is a serious clinical problem. Complications associated with osseous metastases are pain, fractures, spinal cord compression, paralysis, etc., which lead to a significant reduction in the quality of life of tumor patients. Several diagnostic tools are available to detect bone metastases, including plain radiography, computer tomography, magnetic resonance imaging and bone scintigraphy. Of these, radionuclide bone scanning using Tc-99m labeled diphosphonates is the most widely used and accepted method for the detection of bone metastases. Bone scintigraphy can detect osseous metastases several months before changes in plain radiographs can be seen, thus making bone scintigraphy an excellent diagnostic tool. However, this method is expensive, is not always available in every hospital and has the disadvantage of showing a positive reaction even to bone inflammation, degenerative changes and fractures or flare reaction, which leads to a reduced specificity. This is the reason why many authors have described outcomes regarding diagnosis of bone metastases and observation of the clinical course using markers of bone turnover.

In recent years, there have been important advances in the field of biochemical markers of bone turnover and new methods have emerged [8]. Measurement of the metabolites of type I collagen, the predominant collagen in bone, has been reported to be useful for monitoring bone turnover in many different disorders, including diseases with bone metastases [9].

Yoshida et al. reported on the serum concentration of type I collagen metabolites as a quantitative marker of bone metastases in patients with prostate cancer [10]. They concluded that, especially in patients with high grade carcinoma cells, the determination of bone metabolic markers should be more useful in evaluating metastatic spread to bone than prostate specific antigen. In our collective, patients with prostate cancer (n = 5) showed the highest correlation between the presence of osseous metastases and elevated markers for PINP and ICTP. Only in one case we found a false positive ICTP. However, due to the small size of our patient groups it prevents any real conclusive statements from being made.

For other tumor types we did not observe a similar high correlation. Horiguchi et al. reported on the usefulness of ICTP as a marker for bone metastases in patients with lung cancer [11]. He suggested that measurement of ICTP is an excellent serological diagnostic method for identifying bone metastases in patients with lung cancer and can also help predict when it might be useful to undertake other examinations like bone scintigraphy. In our study however, we saw a high rate (11 out of 19) of false positive results of increased ICTP levels in this group of patients. One reason for this might be the presence of non-detectable micro-metastases at the time of ICTP measurement. To avoid the possibility of false results, all patients had a follow-up examination between 9 and 14 months, during which micro-metastases would have become apparent. None of the 11 patients developed osseous metastases during this time period so that the likelihood of the presence of such metastases was very low.

Another major group of patients in our study were females with breast cancer. The literature on these tumors and the value of bone metabolic markers for detection of osseous metastases is very controversial. Blomqvist et al. reported a positive and significant correlation between ICTP and PICP and the number of bone metastases, plus Wada et al. suggested that ICPT might be a useful marker for screening and monitoring bone metastases in breast cancer [12,13]. In contrast Ulrich et al. showed in a study with 106 patients that the sensitivity for diagnosing bone metastases was 65% [14]. These results are more similar to our study. However, Ulrich reported a high specificity of 91%, whereas we observed only 25% specificity for ICTP.

The types of tumors in our study were very heterogeneous, which seems to reflect the circumstances seen on a daily basis. Due to the fact that tumors can metastasize to bone in different ways (osteoblastic and/or osteolytic) we established parameters for both possibilities. For PINP, the marker for osteoblastic activity, the specificity was high but with a poor sensitivity. For ICTP, the marker for osteolytic activity, both characteristics of sensitivity and specificity were low, such that a general recommendation for the use of this marker as a screening parameter cannot be made.

Potential indications for bone metabolic markers might be the therapy control in patients with bone metastases, in which increased parameters had been proven before therapy. Particularly in these patients, the so-called "bone flare reaction", the repair of destroyed bone structures by tumor cells, complicates the assessment of bone scintigraphy. Increased activity in known osseous metastases after or during therapy can be caused by either repair or further tumor growth. Blomqvist and colleagues and Koizumi et al. reported on patients with breast cancer and osseous metastases, where only patients with progressive disease showed an increase in ICTP values during therapy compared to other patients with response to therapy [9,15]. Another indication might be in benign bone disorders like rheumatoid arthritis or Paget's disease. Aman et al. reported a correlation between ICTP values and the disease progression of patients with rheumatoid arthritis [16].

## Conclusions

In summary, when a new method is recommended for the diagnostic work up, it is necessary to demonstrate that this new modality is as sensitive and specific as existing routine imaging procedures. The determination of bone metabolic parameters like ICTP or PINP is less expensive than bone scanning, but this prospective study has shown that the results from bone metabolic markers are not yet sufficient enough to demonstrate a bone involvement in different type of malignancies with high sensitivity and specificity.

## Competing interests

The author(s) declare that they have no competing interests.

## Authors' contributions

JS and CE designed the study, performed the statistical analysis and drafted the manuscript.

SR and EW recruited patients and carried out the nuclear medicine investigations.

All authors read and approved the final manuscript.

**Figure 6:**
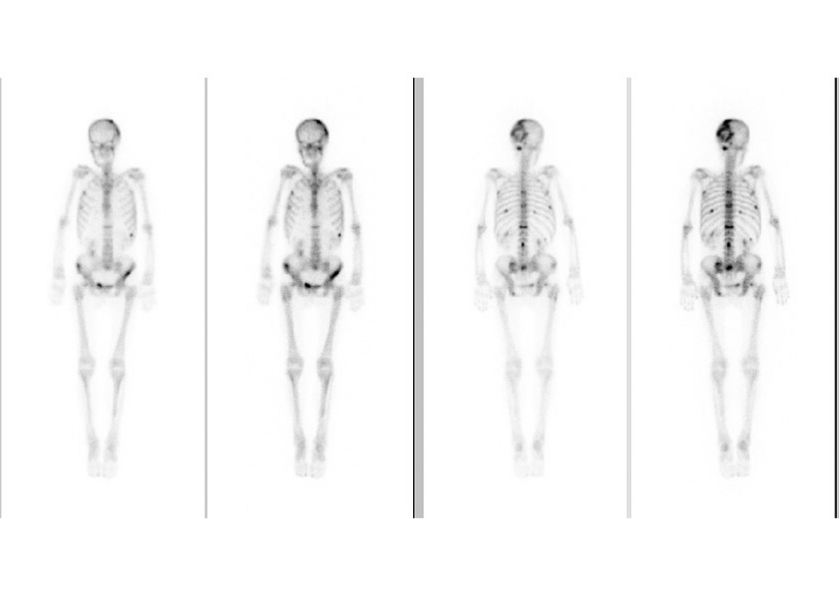
Bone scan of a 59-year-old female with non-small-cell lung cancer and multiple osseous metastases. Both parameters are increased ICTP: 13 μg/L (1.6–5.3) PINP: 113.8 μg/L (19–102)

**Figure 7:**
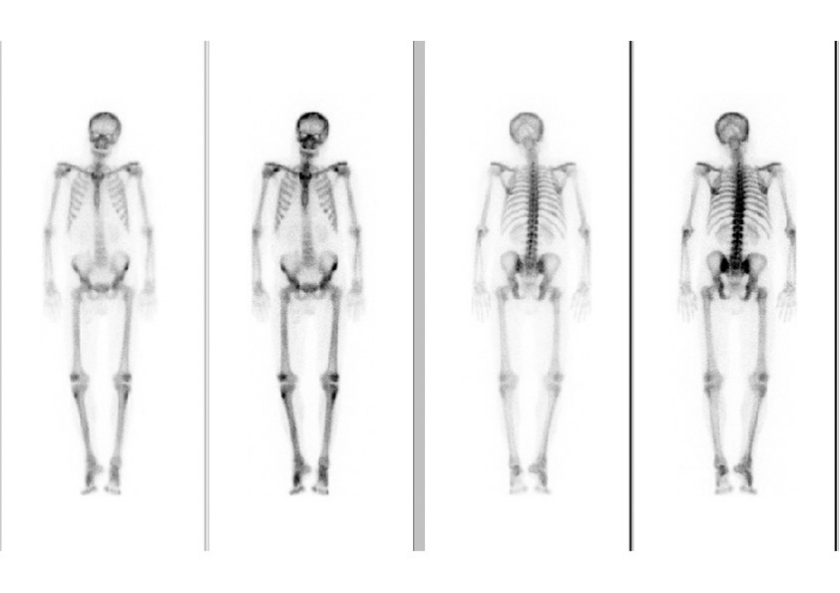
Bone scan of a 47-year-old male with non small cell lung cancer. Bone scintigraphy and follow-up showed no evidence of osseous metastatic disease. ICTP: 7.3 μg/L (1.4–5.2) and PINP: 102 μg/L (21–78) are above the upper reference limit.

**Figure 8:**
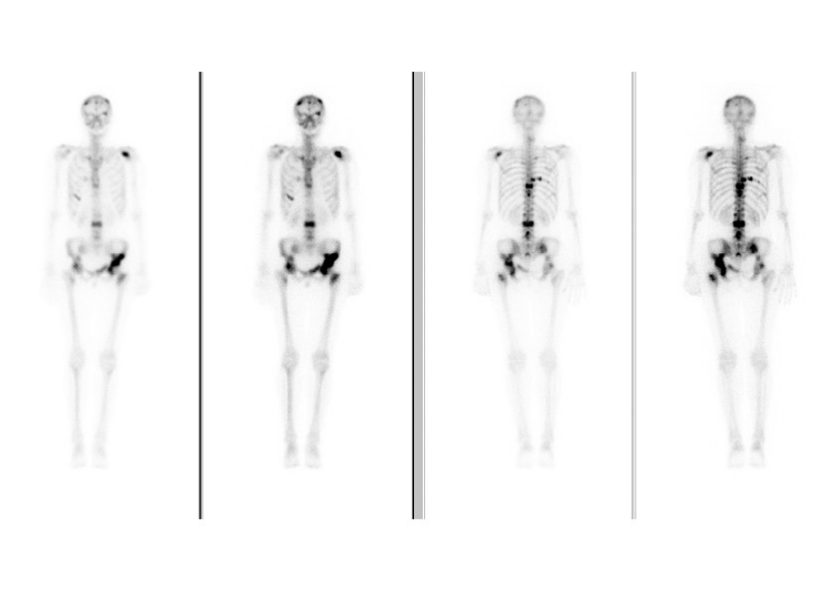
55-year-old female with metastatic breast cancer. On the bone scan multiple osseous metastases can be seen especially in the spine, pelvic region and calotte. ICTP: 4.8 μg/L (1.6–5.3) and PINP: 31.5 μg/L (19–102) are within the reference limit.

## Pre-publication history

The pre-publication history for this paper can be accessed here:


